# Integrating nutrition and lifestyle: associations between employment type and health behaviors

**DOI:** 10.3389/fnut.2026.1889959

**Published:** 2026-10-16

**Authors:** Kai Yao, Wei Chen, Chenxi Bao, Yujie Shi

**Affiliations:** 1 School of Business Administration, Xinjiang University of Science and Technology, Korla, China; 2 Rattana Bundit University, Bangkok, Thailand; 3 Yunnan Saiwei Intelligent Environmental Protection Technology Co., Ltd., Kunming, China; 4 INTI International University, Nilai, Malaysia

**Keywords:** dietary diversity, health outcomes, heterogeneity, lifestyle behaviors, ordered probit model, self-employment

## Abstract

**Introduction:**

Health and well-being are associated with socioeconomic conditions, nutrition, and lifestyle behaviors. Although self-employment has become increasingly prevalent in developing economies, its implications for health remain unclear, particularly through nutrition- and lifestyle-related pathways. This study examines the associations between employment arrangements and self-rated health, dietary diversity, and lifestyle behaviors in Thailand, with particular attention to dietary behaviors.

**Methods:**

Data were drawn from the Thailand Health and Welfare Survey. Ordered probit models and an instrumental variable approach were employed to examine the associations between self-employment and self-rated health, dietary diversity, physical activity, smoking, and alcohol consumption. Heterogeneity across types of self-employment was also assessed by distinguishing employer self-employed individuals from solo self-employed workers.

**Results:**

Self-employment was associated with lower self-rated health and lower dietary diversity, indicating less varied diets. Self-employed individuals also exhibited less favorable lifestyle behaviors, including lower physical activity and higher probabilities of smoking and alcohol consumption. These associations varied by type of self-employment. Employer self-employed individuals tended to maintain healthier behaviors and better health outcomes, whereas solo self-employed workers were more vulnerable to lower dietary diversity and adverse health conditions.

**Discussion:**

The findings indicate that employment type is an important structural factor associated with nutrition, lifestyle behaviors, and health inequalities. Policies aimed at improving population health should integrate labor-market interventions with nutrition- and lifestyle-oriented strategies, with particular attention to vulnerable groups of solo self-employed workers.

## Introduction

Health and wellbeing are central to sustainable development and have become increasingly important in both policy and academic debates. The United Nations' *Transforming Our World: The 2030 Agenda for Sustainable Development* identifies health and wellbeing as a central component of sustainable development (2), while the Healthy Thailand 2030 Outline highlights the need for integrated strategies that address lifestyles, living and working environments, and healthcare systems. In Thailand, rapid industrialization and the expansion of digital and platform-based work have reshaped labor market structures and daily routines. Within this context, self-employment has become an increasingly prevalent form of work (3). Compared with paid employment, it is often characterized by income instability, weaker labor protection, and greater exposure to market risks, and is therefore frequently discussed as a vulnerable employment form (4). Understanding how self-employment is associated with health is thus relevant for explaining labor-market-related health inequalities (5).

Health is a multidimensional concept that goes beyond the absence of disease and includes physical, mental, and social wellbeing. In empirical studies, self-rated health is widely used as a comprehensive indicator because it captures multiple dimensions of individual wellbeing, including physical condition, psychological status, and social functioning (6). Existing research has examined health determinants at both macro and micro levels, including economic development, healthcare systems, income, education, social environment, and lifestyle behaviors (7, 8). However, scholars have reported divergent findings on the roles of absolute and relative income (9–11), social capital (12, 13), housing conditions, internet use, commuting, and healthy habits (14, 15), as well as demographic factors such as gender, age, and education (16).

A growing body of occupational-health research has examined how employment type relates to health risks, wellbeing, and work intensity. Earlier studies linked employment arrangements and self-employment to health indicators and occupational risks (17–20), while more recent longitudinal studies have investigated the health implications of self-employment and employment transitions (21, 22). The evidence remains mixed. On one hand, self-employment may improve wellbeing through greater autonomy, decision-making power, job control, and flexibility (23, 24). On the other hand, self-employed workers may face income uncertainty, longer or irregular working hours, heavier workloads, and occupational stress, which can undermine physical and mental health (25–27). These mixed findings suggest that the relationship between self-employment and health may depend on job characteristics and within-group heterogeneity.

Employment type should therefore not be understood merely as a labor market category, but also as a social determinant of health. The World Health Organization defines social determinants of health refer to the conditions in which people are born, grow, live, work, and age (28). Work conditions may influence health through material resources, psychosocial stress, access to healthcare, and health-related behaviors. Socioeconomic disadvantage and unstable working conditions may contribute to unhealthy behaviors and chronic disease risks (29), while Employment status, job insecurity, work intensity, and lack of labor protection are important sources of health inequality (30). Recent research further suggests that adverse social and employment conditions may be associated with poorer nutrition behaviors, reduced physical activity, and other unhealthy coping behaviors (31).

Nutrition is a particularly important pathway linking employment arrangements and health. Dietary behaviors are embedded in daily routines, time allocation, social position, and stress exposure (32). Studies in public health nutrition show that shift work, long working hours, job strain, and occupational stress are often associated with lower diet quality, reduced fruit and vegetable intake, higher consumption of energy-dense or processed foods, and more frequent out-of-home eating (33–35). Irregular work schedules may also disrupt meal timing and dietary regularity, contributing to meal skipping, unhealthy food choices, and lower dietary quality (36). From an occupational health perspective, worksite and employment conditions can either facilitate or constrain healthy eating, and multicomponent interventions that integrate nutrition, physical activity, stress management, and behavioral support may improve health more effectively than single-focus interventions (37).

Beyond nutrition, self-employment may also be associated with other lifestyle-related behaviors, including physical activity, smoking, alcohol consumption, sleep patterns, and healthcare utilization. High job demands and long working hours may reduce leisure time and limit physical activity (38), while job insecurity and psychosocial stress may increase unhealthy coping behaviors such as smoking and alcohol consumption (39). Self-employment combines autonomy with economic uncertainty: flexibility may enable some individuals to manage their health more effectively, whereas irregular hours, continuous work engagement, and limited institutional support may reduce exercise, disrupt sleep, delay preventive care, and weaken health-related investment (30, 40). Such risks may be especially pronounced among solo self-employed workers, who often have fewer resources, weaker protection, and greater market exposure than employer self-employed individuals (27).

Despite these insights, several gaps remain. First, previous studies have often treated self-employment as part of informal employment without fully clarifying its specific relationship with health. Following the conceptual framework of informal employment, this study treats self-employment as a distinct labor market status that includes both employers and own-account workers within the informal employment sector (41). Second, prior research has not sufficiently examined nutrition and lifestyle-related correlates of self-employment, especially dietary diversity, physical activity, smoking, and alcohol consumption. Third, endogeneity concerns, including omitted variables and reverse causality from health to employment choice, require cautious empirical discussion. Using data from the 2024 Thailand Health and Welfare Survey, this study examines the association between self-employment and self-rated health, explores nutrition and lifestyle-related correlates, and distinguishes between employer self-employment and solo self-employment. The study does not directly observe motivations for entering self-employment; therefore, interpretations related to voluntary or necessity-driven self-employment are beyond its scope (27).

Heterogeneity within self-employment is central to this study. Employer self-employed individuals and solo self-employed workers differ in resources, income stability, working conditions, and exposure to market risks. Self-employed workers with employees may have greater control over work organization and income streams, whereas solo self-employed individuals may face higher income volatility and lower health-related investment capacity (40). From a nutrition and lifestyle perspective, these differences may translate into unequal capacities to maintain balanced diets, regular meal patterns, physical activity, and preventive health behaviors.

Based on the above, we propose three hypotheses:

Hypothesis 1: Self-employment is negatively associated with residents' health outcomes.

Hypothesis 2: Lifestyle-related constraints, including irregular work schedules, limited health investment, and unhealthy behavioral patterns, constitute potential links between self-employment and health outcomes.

Hypothesis 3: The health association of self-employment differs between employer self-employment and solo self-employment. Employer self-employment is more likely to be associated with better health, whereas solo self-employment is more likely to be associated with poorer health.

## Materials and methods

### Data source

This study uses data from the 2024 Thailand Health and Welfare Survey. The Thailand Health and Welfare Survey (HWS) employed a nationally representative multi-stage stratified cluster sampling design. To account for the complex survey design, all analyses incorporated the sampling weights provided by the HWS. Standard errors were estimated using Taylor-series linearization, with stratification and primary sampling units specified according to the survey design. The survey covers domains such as education, employment, health, and medical insurance. The initial sample included all respondents in the working-age population. The final analytical sample was restricted to individuals aged 18–65 years. Observations with missing values in key variables (self-rated health, employment status, and core covariates) were excluded using listwise deletion. In addition, extreme outliers in continuous variables (income and working hours) were identified and removed based on the 1st and 99th percentile trimming rule. After applying these exclusion criteria, the final sample consisted of 5,287 observations.

The dependent variable, self-rated health is the most widely adopted indicator for measuring health in empirical studies (42, 43). This measure captures both physical and mental health and is widely regarded as comprehensive and robust (44). Accordingly, this study adopts the questionnaire item: “Compared with people of the same age, how would you assess your current health outcomes?” Scores from 1 to 5 are assigned to the five Likert scale levels, respectively: very poor, poor, fair, good, and very good.

In terms of the core explanatory variable, the Organization for Economic Co-operation and Development (OECD) defines self-employed workers as those who make independent business decisions, bear profits and losses, and derive income from their own business accounts. Following previous classification criteria, this study divides self-employed individuals into employers and solo self-employed workers according to whether they hire paid employees (45, 46). The HWS questionnaire classifies employment status into nine categories. This paper defines employers and own-account workers as self-employed and assigns them a value of 1. Long-term employees, contract workers, hourly workers, and paid domestic workers are categorized as non-self-employed and assigned a value of 0. To accurately examine the association between self-employment and health and mitigate omitted variable bias, a set of control variables was selected based on prior studies, covering personal characteristics, regional attributes, economic conditions, and healthcare factors.

Although the main empirical analysis focuses on self-rated health and short-term morbidity, this study interprets employment type as a structural factor associated with lifestyle conditions. The Thailand Health and Welfare Survey provides an appropriate data source because it integrates information on employment, health, nutrition, and household socioeconomic characteristics.

Healthcare utilization was coded as 1 if respondents reported receiving healthcare services during the reference period and 0 otherwise. Therefore, the low prevalence reflects the limited proportion of respondents requiring healthcare during the survey period rather than limited healthcare accessibility.

Dietary diversity is constructed as a count-based measure of food group consumption and follows standard dietary diversity scoring approaches widely used in nutritional epidemiology, particularly the FAO Household Dietary Diversity Score (HDDS) framework. This allows comparability with existing nutrition and public health literature.

### Inclusion and exclusion criteria

The following inclusion criteria were applied: (1) respondents aged 18–65 years, defined as the working-age population; (2) respondents with valid information on employment status; and (3) respondents who participated in the 2024 Thailand Health and Welfare Survey.

Observations were excluded if they met any of the following criteria: (1) age outside the 18–65 range; (2) missing values on key variables (self-rated health, employment status, and core covariates), handled via listwise deletion; and (3) extreme outliers in continuous variables (monthly income and weekly working hours), identified and removed using the 1st and 99th percentile trimming rule. After applying these criteria, the final analytical sample comprised 5,287 observations.

### Variables and measurement

#### Dependent variable

Self-rated health was measured using the questionnaire item “Compared with people of the same age, how would you assess your current health?” Responses were recorded on a five-point ordinal Likert scale (1 = very poor, 2 = poor, 3 = fair, 4 = good, 5 = very good).

#### Core independent variable

Self-employment was a binary indicator (= 1 if the respondent was classified as an employer or own-account worker; = 0 if the respondent was a long-term employee, contract worker, hourly worker, or paid domestic worker). For heterogeneity analysis, self-employment was further disaggregated into employer self-employment (= 1 if hiring paid employees) and solo self-employment (= 1 if no paid employees), both binary.

#### Control variables

The following covariates were included: gender (binary; male = 1), age group (categorical; 18–25, 26–35, 36–45, 46–55, 56–65), years of education (continuous), residence (binary; urban = 1), region (categorical; eastern, central, western, southern, northern), monthly income (continuous; Thai baht, rescaled in thousands), weekly working hours (continuous), medical insurance (binary; insured = 1), and healthcare utilization (binary; = 1 if healthcare services were received during the reference period).

#### Nutrition and lifestyle variables

Dietary diversity was measured as a count score ranging from 0 to 10, constructed from the 24-h dietary recall module by summing consumption across 10 food groups: cereals and staples, roots and tubers, vegetables, fruits, meat and poultry, fish and seafood, eggs, legumes and nuts, dairy products, and oils and fats. The measure follows an adapted version of the FAO Household Dietary Diversity Score framework and should be interpreted as an indicator of dietary variety rather than a comprehensive measure of nutritional status. Physical activity was coded as a binary variable equal to 1 if the respondent reported regular physical activity and 0 otherwise. Smoking was coded as 1 for current smokers and 0 otherwise. Alcohol consumption was coded as 1 for current drinkers and 0 otherwise. Because the dietary diversity measure was derived from a single 24-h dietary recall, it may not reflect usual intake and does not capture energy intake, nutrient adequacy, portion size, or overall dietary quality.

#### Objective health indicators (robustness checks)

Five binary indicators captured short-term morbidity over the past 4 weeks: fever/sore throat/cough, diarrhea, stomachache, headache/dizziness, and joint/muscle pain (= 1 if the symptom was experienced). A composite monthly morbidity indicator was also used (= 1 if any symptom was reported).

#### Instrumental variable

The regional urban registered unemployment rate (continuous; percentage), derived from the Thailand Statistical Yearbook, served as the instrument for self-employment in the IV sensitivity analysis.

Detailed variable definitions and coding are provided in Supplementary Table S1.

### Statistical analysis

All analyses were conducted in Stata/MP 16.0. Because the HWS employed a multi-stage stratified cluster sampling design, all estimates incorporated sampling weights, stratification, and primary sampling units; standard errors were estimated using Taylor-series linearization. Survey design features were implemented using the “svy” suite of commands; ordered probit models were estimated using the “oprobit” command with survey-adjusted variance estimation.

#### Descriptive statistics

Means, standard deviations, and proportions were computed for the full sample and by employment subgroup.

#### Baseline model

The association between self-employment and self-rated health was estimated using a survey-adjusted ordered probit model:


$$\begin{array}{l} {\text{y}}^{*}=\text{β}0+\text{β}1\text{SelfEmployment}+\text{βXX}+\text{ ε} \end{array}$$

where y represents self-rated health (1–5 ordinal), Self Employment is a binary indicator, and X includes demographic, socioeconomic, regional, and healthcare controls. The reference category was non-self-employed workers.

Previous studies have adopted variables such as the number of self-employed family members, migration status, and children's insurance coverage as instrumental variables (47). This sensitivity analysis is implemented within a control-function framework for a binary endogenous regressor, consistent with a control-function approach for a binary endogenous regressor (48). This paper selects the regional urban registered unemployment rate derived from the Thailand Statistical Yearbook as the instrumental variable for self-employment. In regions with high unemployment rates, unemployed individuals tend to turn to self-employment due to resource constraints. Rural migrant workers also struggle to secure local jobs and choose self-employment to maximize household utility. Hence, the urban unemployment rate is positively correlated with self-employment. Meanwhile, the validity of the instrumental variable relies on the assumption that regional unemployment affects individual health primarily through its impact on self-employment decisions. The theoretical justification for this exclusion restriction is based on labor market allocation mechanisms: higher regional unemployment increases entry into self-employment due to constrained wage employment opportunities, particularly in informal or small-scale economic activities. While regional unemployment may be correlated with broader economic conditions, its direct effect on individual health is expected to be limited after controlling for individual socioeconomic characteristics, regional fixed effects, and healthcare access variables included in the model. Nevertheless, the exclusion restriction underlying the instrumental variable strategy should be interpreted with caution. Regional unemployment rates may influence individual health outcomes through channels other than employment status. For example, regional economic conditions may affect income opportunities, food availability, and price levels, thereby shaping dietary behaviors and nutrition quality. In addition, differences in local healthcare infrastructure and access to medical services may directly influence health outcomes. These potential alternative pathways cannot be fully ruled out in the present analysis. Given that regional unemployment may influence health through multiple channels—including local economic conditions, food environments, and healthcare infrastructure—the exclusion restriction is likely violated. Therefore, the IV results should be interpreted strictly as descriptive sensitivity checks and not as causal estimates. The IV analysis is included only to assess whether the baseline association persists under an alternative specification. Therefore, conditional on these controls, regional unemployment is assumed to influence health outcomes mainly through employment status rather than through independent pathways.

To evaluate instrument relevance, we report first-stage coefficients and first-stage *F* statistics. Following the conventional rule of thumb, the instrument shows sufficient predictive power for self-employment. Standard errors are clustered at the regional level to account for within-region correlation.

To further assess instrument strength, we report first-stage *F*-statistics under progressively expanded specifications. All values exceed the conventional threshold of 10, suggesting that the instrument is not weak according to the conventional weak-instrument rule of thumb (49). In addition, we estimate the first-stage models under survey-weighted specification with clustering at the regional level to account for within-region correlation in unobserved shocks. The use of Taylor linearization ensures that standard errors properly reflect the complex survey design. While alternative weak-instrument robust statistics such as Kleibergen–Paap are not directly applicable under the survey-adjusted ordered probit framework, the consistently high *F*-statistics provide reassuring evidence of instrument relevance.

#### Marginal effects

Average marginal effects (AMEs) were computed at sample means to quantify the probability shift across each health category.

#### Heterogeneity analysis

Separate ordered probit models were estimated for employer self-employed and solo self-employed subsamples, each compared against non-self-employed workers.

#### Exploratory lifestyle-correlate analysis

To explore whether self-employment is associated with lifestyle-related factors, we examined its associations with dietary diversity, physical activity, smoking, and alcohol consumption. Dietary diversity (continuous count score) was modeled with survey-adjusted OLS; binary lifestyle outcomes were modeled with survey-adjusted probit regressions. These analyses were intended to identify potential correlates rather than establish mediation or causal pathways. Missing data were addressed using listwise deletion; the proportion of missingness for all variables was below 5%, and no imputation was applied.

#### IV sensitivity analysis

To address as a sensitivity check for potential endogeneity from reverse causality and omitted variable bias, an instrumental-variable analysis was conducted within a control-function framework for the binary endogenous regressor (48). The regional urban registered unemployment rate was used as the excluded instrument. First-stage *F*-statistics were reported to assess instrument relevance (*F* > 10 as the threshold).

#### Robustness checks

Two sets of checks were performed: (i) re-estimation using OLS and IV-2SLS; (ii) replacement of the dependent variable with objective short-term morbidity indicators, estimated using binary probit models.

Significance was evaluated at *p* < 0.10, *p* < 0.05, and *p* < 0.01.

## Results

### Participant characteristics

Sample characteristics and distributions are presented in Table 1.

**Table 1:** Descriptive statistical analysis of samples.

Variables	Full sample	Self-employed	Employers	Solo self-employed	Non-self-employed
Sample proportion (%)	100.00	39.08	9.29	29.79	60.92
Gender (%)					
Male	57.44	59.49	56.82	60.32	56.13
Female	42.56	40.51	43.18	39.68	43.87
Age (years)	43.52	45.37	41.90	46.45	42.33
Age group (%)					
18–25	5.31	3.87	6.31	3.11	6.24
26–35	19.50	15.83	24.85	13.02	21.86
36–45	29.15	28.41	29.12	28.19	29.62
46–55	31.74	32.33	28.31	33.59	31.36
56–65	14.30	19.55	11.41	22.10	10.93
Years of education	11.07	9.24	10.47	8.86	12.24
Education level (%)					
Primary or below	13.90	24.39	13.24	27.87	7.17
Junior high school	35.98	49.52	44.81	50.98	27.29
Senior high/vocational	26.90	20.76	28.11	18.48	30.83
College or above	23.23	5.32	13.85	2.67	34.71
Health insurance (%)					
Yes	97.96	97.63	96.95	97.84	98.17
No	2.04	2.37	3.05	2.16	1.83
Healthcare utilization (%)					
Yes	2.72	1.94	1.63	2.03	3.23
No	97.28	98.06	98.37	97.97	96.77
Residence (%)					
Urban	37.19	18.34	30.96	14.41	49.27
Rural	62.81	81.66	69.04	85.59	50.73
Region (%)					
Eastern	23.81	14.33	26.68	10.48	29.90
Western	26.37	32.82	25.87	34.98	22.23
Central	22.15	24.39	29.12	22.92	20.71
Northern	27.67	28.46	18.33	31.62	27.17
Monthly income (Thai baht)	18,470.37	19,068.43	19,394.42	18,966.79	18,086.72
Weekly working hours	42.08	40.73	47.66	38.56	42.95

Any units not labeled in the table are percentages.

In terms of individual characteristics, males constitute 57.4% of the sample and females 42.6%. The majority of respondents are aged 46 to 55 years, with an overall mean age of 43.5 years. Among the self-employed, males account for 59.5% and females 40.5%, indicating a slightly higher proportion of male self-employed workers. The average age of the self-employed is 45.4 years, compared with 42.3 years for paid employees. Before age 45, paid employees outnumber the self-employed; after age 45, particularly between 56 and 65 years, the proportion of self-employed workers gradually exceeds that of paid employees. This age distribution aligns with conventional expectations: younger workers tend to take salaried jobs to accumulate human and financial capital, transitioning to self-employment in middle age, whereas middle-aged individuals bearing family responsibilities are either motivated or compelled to engage in self-employment. The average years of schooling for the full sample is 11.07 years. This figure stands at 9.24 years for the self-employed and 12.24 years for paid employees, which is 1.32 times that of the self-employed. Junior high school education is the most prevalent among the self-employed (49.52%), while college education or above ranks first among paid employees (34.71%). Although self-employment might seem to require professional expertise, over 80% of self-employed respondents reside in rural areas where entry barriers are low and basic literacy suffices. Regarding healthcare indicators, 98% of participants have medical insurance coverage, yet only 2.7% have received healthcare services. In terms of regional distribution, 81.7% of the self-employed are rural residents-−4.5 times the urban proportion. Paid employees are more evenly distributed across urban and rural areas, with 49.3% urban and 50.7% rural. Most self-employed workers live in western Thailand, accounting for 32.8%, whereas paid employees are mainly concentrated in eastern Thailand (29.9%). Concerning economic features, the monthly average income of the self-employed is 981.71 Thai baht higher than that of paid employees, while paid workers work 2.22 more hours per week. This suggests that self-employment may be an economically rational choice, offering higher income with shorter working hours. Further analysis reveals that self-employed employers have an average age of 41.9 years, younger than solo self-employed workers (46.5 years). Employers also receive 1.61 more years of education and have a larger share of senior high school and above educational attainment. Moreover, eastern and central regions exhibit a higher ratio of self-employed employers relative to solo self-employed individuals.

### Health outcomes and distribution

As shown in Table 2, the average self-rated health score for the total sample is 3.71, which falls between the fair and good levels. The mean self-rated health score of the self-employed is 3.64, which is lower than that of non-self-employed groups. This difference is statistically significant, indicating that non-self-employed workers report slightly better self-perceived health. Regarding objective health indicators, the self-employed report lower average values for fever, sore throat, cough, and diarrhea over the past 4 weeks. In contrast, they present higher average values for stomachache, headache, dizziness, as well as joint and muscle soreness during the same period. Overall, the self-employed report poorer self-rated health and a higher prevalence of some symptoms compared with non-self-employed employees. Moreover, self-employed employers obtain marginally higher self-rated health scores than solo self-employed individuals, with the difference significant at the 1% level.

**Table 2:** Residents' health outcomes.

Variables		Full sample		Self-employed								Non-self-employed		Difference	
				All		Employers		Solo		Difference					
		Mean	Std. dev.	Mean	Std. dev.	Mean	Std. dev.	Mean	Std. dev.	Solo–employer	*t*-value	Mean	Std. dev.	Non-SE–SE	*t*-value
Subjective indicators		3.707	0.768	3.638	0.764	3.784	0.749	3.592	0.763	−0.192^***^	−4.929	3.752	0.767	0.114^***^	5.272
Objective indicators	Indicator 1	0.060	0.237	0.051	0.220	0.049	0.216	0.051	0.221	0.003	0.227	0.066	0.248	0.015^**^	2.256
	Indicator 2	0.011	0.105	0.010	0.098	0.012	0.110	0.009	0.094	−0.003	−0.606	0.012	0.109	0.002	0.840
	Indicator 3	0.017	0.131	0.020	0.141	0.018	0.134	0.021	0.143	0.003	0.372	0.016	0.124	−0.005	−1.267
	Indicator 4	0.024	0.151	0.024	0.154	0.008	0.090	0.029	0.168	0.021^**^	3.585	0.023	0.150	−0.001	−0.286
	Indicator 5	0.029	0.169	0.033	0.180	0.022	0.148	0.037	0.188	0.014^*^	1.759	0.027	0.161	−0.007	−1.376

(1) The subjective health indicator is self-rated health, measured on a five-point scale ranging from 1 (very poor) to 5 (excellent).

(2) Objective health indicators capture short-term physical conditions. Specifically: Indicator 1 refers to fever, sore throat, or cough in the past 4 weeks; Indicator 2 refers to diarrhea; Indicator 3 refers to stomachache; Indicator 4 refers to headache or dizziness; Indicator 5 refers to joint or muscle pain. All indicators are binary variables equal to 1 if the respondent experienced the symptom in the past 4 weeks, and 0 otherwise.

The symbols ^*^, ^**^, and ^***^ indicate statistical significance at the 10%, 5%, and 1% levels, respectively. Specifically, ^*^p < 0.10, ^**^p < 0.05, and ^***^p < 0.01.

### Benchmark regression results and analysis

Table 3 presents the ordered probit estimation results. Model 1 includes only regional controls; the coefficient for self-employment is negative and significant at the 1% level. Model 2 adds individual, healthcare, and economic controls; the coefficient remains negative and significant at the 10% level, with an increase in Pseudo R^2^.

**Table 3:** Oprobit estimation results of self-employment on self-rated health (survey-adjusted estimates).

Variables	All		Self-employed individuals with employees		Self-employed individuals without employees	
	Model 1	Model 2	Model 3	Model 4	Model 5	Model 6
Self-employment (= 1)	−0.095^***^ (0.033)	−0.061^*^ (0.033)	0.139^***^ (0.051)	0.115^**^ (0.052)	−0.173^***^ (0.035)	−0.126^***^ (0.036)
	[−0.160, −0.030]	[−0.126, 0.004]	[0.039, 0.239]	[0.013, 0.217]	[−0.242, −0.104]	[−0.197, −0.055]
Gender		0.048 (0.031)		0.049 (0.031)		0.048 (0.031)
		[−0.013, 0.109]		[−0.012, 0.110]		[−0.013, 0.109]
Age group (ref: 18–25)						
26–35		−0.125^*^		−0.128^*^		−0.122^*^
		(0.072)		(0.072)		(0.072)
36–45		−0.304^***^		−0.308^***^		−0.295^***^
		(0.070)		(0.070)		(0.070)
46–55		−0.375^***^		−0.379^***^		−0.364^***^
		(0.069)		(0.070)		(0.070)
56–65		−0.529^***^		−0.540^***^		−0.509^***^
		(0.077)		(0.077)		(0.077)
Health insurance		−0.048 (0.120)		−0.035 (0.120)		−0.051 (0.121)
		[−0.283, 0.187]		[−0.270, 0.200]		[−0.288, 0.186]
Access to healthcare		−0.201^*^ (0.104)		−0.193^*^ (0.104)		−0.201^*^ (0.104)
		[−0.405, 0.003]		[−0.397, 0.011]		[−0.405, 0.003]
Residence (urban = 1)	−0.006 (0.032)	−0.005 (0.033)	0.027 (0.031)	0.017 (0.032)	−0.026 (0.033)	−0.021 (0.033)
	[−0.069, 0.057]	[−0.070, 0.060]	[−0.034, 0.088]	[−0.046, 0.080]	[−0.091, 0.039]	[−0.086, 0.044]
Region (ref: Eastern)						
Western	−0.567^***^ (0.043)	−0.592^***^ (0.044)	−0.588^***^ (0.042)	−0.606^***^ (0.043)	−0.546^***^ (0.043)	−0.575^***^ (0.044)
	[−0.651, −0.483]	[−0.678, −0.506]	[−0.670, −0.506]	[−0.690, −0.522]	[−0.630, −0.462]	[−0.661, −0.489]
Central	−0.280^***^ (0.045)	−0.302^***^ (0.045)	−0.301^***^ (0.044)	−0.317^***^ (0.044)	−0.268^***^ (0.045)	−0.292^***^ (0.045)
	[−0.368, −0.192]	[−0.390, −0.214]	[−0.387, −0.215]	[−0.403, −0.231]	[−0.356, −0.180]	[−0.380, −0.204]
Northern	−0.065 (0.044)	−0.069 (0.045)	−0.075^*^ (0.044)	−0.075^*^ (0.044)	−0.045 (0.045)	−0.054 (0.045)
	[−0.151, 0.021]	[−0.157, 0.019]	[−0.161, 0.011]	[−0.161, 0.011]	[−0.133, 0.043]	[−0.142, 0.034]
Monthly income (× 1,000 THB)		0.000 (0.000)		0.000 (0.000)		0.000 (0.000)
		[0.000, 0.000]		[0.000, 0.000]		[0.000, 0.000]
Weekly working hours		−0.000 (0.001)		−0.000 (0.001)		−0.000 (0.001)
		[−0.002, 0.002]		[−0.002, 0.002]		[−0.002, 0.002]
Observations	5,287	5,287	5,287	5,287	5,287	5,287
Pseudo *R*^2^	0.0202	0.0279	0.0201	0.0281	0.0215	0.0287

The dependent variable is self-rated health (1 = very poor, 5 = very good). The base category is non-self-employed workers. Models are estimated using survey-adjusted ordered probit regressions incorporating sampling weights, stratification, and clustering. Figures in parentheses are survey-adjusted standard errors. Marginal effects are reported in Table 4. Income variable is rescaled in thousands of Thai baht.

The symbols ^*^, ^**^, and ^***^ indicate statistical significance at the 10%, 5%, and 1% levels, respectively. Specifically, ^*^p < 0.10, ^**^p < 0.05, and ^***^p < 0.01.

For the subsample analyses (Models 3–6), the coefficient for employer self-employment is positive and significant at the 5% level, whereas the coefficient for solo self-employment is negative and significant at the 5% level.

The subgroup analyses compare employer self-employed and solo self-employed individuals against the baseline group of non-self-employed workers. The results are consistent with Hypothesis 1. Self-employment is significantly associated with lower levels of self-rated health. This pattern suggests a negative association between self-employment and health outcomes, but it should not be interpreted as a definitive causal effect.

### Marginal effect analysis

Table 4 presents the average marginal effects. At sample means, self-employment increases the probability of rating health as poor by 0.4% and fair by 1.78%, while decreasing the probability of good health by 0.90% and very good health by 1.36%. For employer self-employed individuals, the probabilities of very poor, poor, and fair health decrease by 0.07, 0.84, and 3.35%, respectively, while good and very good health increase by 1.69 and 2.57%. For solo self-employed workers, the probabilities of very poor, poor, and fair health increase by 0.08, 0.92, and 3.67%, while good and very good health decrease by 1.86 and 2.81%.

**Table 4:** Marginal effects of self-employment on residents' self-rated health.

Self-rated health	Full sample	Employers	Solo self-employed
Very poor	0.0004	−0.0007^*^	0.0008^**^
Poor	0.0044^*^	−0.0084^**^	0.0092^***^
Fair	0.0178^*^	−0.0335^**^	0.0367^***^
Good	−0.0090^*^	0.0169^**^	−0.0186^***^
Very good	−0.0136^*^	0.0257^***^	−0.0281^***^

This table reports average marginal effects (AMEs) from survey-adjusted ordered probit models. Standard errors are not available for marginal effects in the original output; therefore, 95% confidence intervals are not reported. All models include sampling weights, stratification, and regional clustering using Taylor-series linearization. The reference category is non-self-employed workers. Sample size is *N* = 5,287. Missing data are handled using listwise deletion. The symbols ^*^, ^**^, and ^***^ indicate statistical significance at the 10%, 5%, and 1% levels, respectively. Specifically, ^*^p < 0.10, ^**^p < 0.05, and ^***^p < 0.01.

### Exploratory analysis of lifestyle-related correlates

#### Nutrition pathways

Employment conditions may influence nutritional outcomes through income stability, time constraints, food environment accessibility, and work-related stress, all of which are key determinants in nutrition science.

Table 5 presents the associations between self-employment and lifestyle-related variables. Self-employment is negatively associated with dietary diversity (coefficient = −0.037, *p* < 0.10) and physical activity (coefficient = −0.045, *p* < 0.05), and positively associated with smoking (coefficient = 0.032, *p* < 0.05) and alcohol consumption (coefficient = 0.028, *p* < 0.10).


$$\begin{array}{c} {Lifestyle}_{i}=\alpha +\beta {selfEmployment}_{i}+{yX}_{i}+\varepsilon_{i} \end{array}$$ (1)

**Table 5:** Mechanism analysis: lifestyle behaviors.

Variables	Dietary diversity	Physical activity	Smoking	Drinking
Self-employment	−0.037^*^	−0.045^**^	0.032^**^	0.028^*^
	(0.021) [−0.078, 0.004]	(0.019) [−0.082, −0.008]	(0.015) [0.003, 0.061]	(0.016) [−0.003, 0.059]
Controls	Yes	Yes	Yes	Yes
Province FE	Yes	Yes	Yes	Yes
Observations	5,287	5,287	5,287	5,287
Pseudo R^2^	0.065	0.071	0.062	0.058

Each column reports survey-adjusted OLS/probit estimates depending on outcome type. Standard errors are survey-adjusted and clustered at regional level. All models compare self-employed individuals with non-self-employed workers as the reference group.

The symbols ^*^, ^**^, and ^***^ indicate statistical significance at the 10%, 5%, and 1% levels, respectively. Specifically, ^*^p < 0.10, ^**^p < 0.05, and ^***^p < 0.01.

### Lifestyle pathway (non-dietary behaviors)

To further examine the potential lifestyle pathway, this study introduces several lifestyle-related variables available in the HWS, including smoking, alcohol consumption, physical activity, and dietary diversity. The results show that self-employment is associated with differences in lifestyle behaviors. Specifically, self-employed individuals tend to report less favorable lifestyle patterns and health investment behaviors.

Beyond nutrition, self-employment also associated with other lifestyle behaviors, including physical activity, smoking, and alcohol consumption. Although not all coefficients reach conventional levels of statistical significance, the overall pattern is consistent with the theoretical expectation. These findings are consistent with Hypothesis 2 and suggest that lifestyle-related factors may be relevant correlates of both self-employment and health outcomes. However, given the cross-sectional nature of the data, these results should be interpreted as exploratory associations rather than evidence of mediation or causal pathways. This pattern is also consistent with the theoretical framework, which suggests that irregular working schedules, time constraints, and economic uncertainty may translate into unhealthy lifestyle behaviors among the self-employed.

### Sensitivity analysis for potential endogeneity

#### Instrumental-variable sensitivity analysis

The following IV results are presented as sensitivity checks only and should not be interpreted causally. Residents' health outcomes may also influence their decision to engage in self-employment. Reverse causality and omitted variables may exist between self-employment and health, leading to endogeneity in model specification. Under current social circumstances, self-employment may attract individuals with poor health, representing a form of passive self-selection. At the same time, it may also draw healthier individuals, who are more capable of seizing business opportunities and accessing financing (50). Therefore, an instrumental-variable specification is reported as a sensitivity analysis to probe the robustness of the baseline association; it is not intended to establish causal inference of the previous regression results.

Table 6 presents the first-stage results. Across four stepwise specifications, the coefficient for the urban unemployment rate remains positive and significant at the 1% level. The first-stage *F*-statistics range from 72.89 to 88.64.

**Table 6:** First-stage regression for the instrumental-variable sensitivity analysis.

Variables	Self-employment			
	(1)	(2)	(3)	(4)
Urban unemployment rate	0.177^***^	0.171^***^	0.167^***^	0.167^***^
	(0.024) [0.130, 0.224]	(0.024) [0.124, 0.218]	(0.024) [0.120, 0.214]	(0.024) [0.120, 0.214]
Individual characteristics	No	Yes	Yes	Yes
Healthcare characteristics	No	No	Yes	Yes
Economic characteristics	No	No	No	Yes
Regional characteristics	Yes	Yes	Yes	Yes
Constant	−1.061^***^	−1.398^***^	−1.001^***^	−1.000^***^
	(0.094)	(0.127)	(0.179)	(0.187)
Observations	5,287	5,287	5,287	5,287
Pseudo R^2^	0.1138	0.1268	0.1285	0.1299
*F*-statistic (excluded instrument)	72.89	81.85	86.74	88.64

Standard errors are reported in parentheses. ^***^p < 0.01, ^**^p < 0.05, ^*^p < 0.1. The model is estimated using survey-adjusted first-stage regressions. The F-statistic of the exclusion tool (urban unemployment rate) is shown in the figure, indicating strong tool correlation and no weak tool correlation. The first-stage F-statistic is reported to assess instrument strength and exceeds the conventional threshold of 10, indicating no weak instrument concern.

#### Sensitivity Analysis for Potential Endogeneity

The IV results are presented as sensitivity checks only and should not be interpreted as causal estimates. Table 6 reports the first-stage results. Across the four stepwise specifications, the coefficient of the regional urban registered unemployment rate is positive and statistically significant at the 1% level. The first-stage *F*-statistics range from 72.89 to 88.64, exceeding the conventional threshold of 10 and suggesting that the instrument is strongly correlated with self-employment.

Table 7 presents the second-stage results. The fitted coefficient for self-employment remains negative and statistically significant at the 1% level across all specifications. The IV-based marginal effects in the fully adjusted model indicate that self-employment is associated with a higher probability of reporting very poor or poor health and a lower probability of reporting good or very good health. These results are directionally consistent with the baseline ordered probit estimates.

**Table 7:** Instrumental-variable sensitivity analysis.

Variables	Health			
	(1)	(2)	(3)	(4)
Predicted self-employment	−0.912^***^	−0.931^***^	−0.999^***^	−1.028^***^
	(0.297) [−1.494, −0.330]	(0.304) [−1.526, −0.336]	(0.308) [−1.603, −0.395]	(0.309) [−1.635, −0.421]
Individual characteristics	No	Yes	Yes	Yes
Healthcare characteristics	No	No	Yes	Yes
Economic characteristics	No	No	No	Yes
Regional characteristics	Yes	Yes	Yes	Yes
Cut1	−3.502	−3.721	−3.922	−3.934
	(0.156)	(0.148)	(0.212)	(0.230)
Cut2	−2.382	−2.599	−2.795	−2.807
	(0.117)	(0.108)	(0.186)	(0.193)
Cut3	−0.816	−1.014	−1.210	−1.221
	(0.113)	(0.103)	(0.182)	(0.189)
cut4	0.509	0.324	0.129	0.118
	(0.113)	(0.103)	(0.182)	(0.189)
*N*	5,287	5,287	5,287	5,287
Pseudo R^2^	0.0203	0.0279	0.0284	0.0286

Standard errors are in parentheses. Ninety-five percent confidence intervals are reported in square brackets. All models include sampling weights, stratification, and regional clustering using Taylor-series linearization. Reference category: non-self-employed workers. Sample size *N* = 5,287. Missing data handled using listwise deletion.

The symbols ^*^, ^**^, and ^***^ indicate statistical significance at the 10%, 5%, and 1% levels, respectively. Specifically, ^*^p < 0.10, ^**^p < 0.05, and ^***^p < 0.01.

#### Robustness test

##### Changing estimation method

Table 8 presents results using OLS and IV-2SLS estimators. The coefficient for self-employment is negative and significant at the 10% level in both models.

**Table 8:** Robustness test I: results of alternative estimation methods.

Variable		OLS	IV-2SLS (first stage)	IV-2SLS (second stage)
Core explanatory variable	Self-employment (= 1)	−0.0416^*^ (0.0228) [−0.086, 0.003]	—	−0.444^*^ (0.247) [−0.928, 0.040]
Instrumental variable	Urban unemployment rate	—	0.057^***^(0.008)	—
Control variables	Individual characteristics	Yes	Yes	Yes
	Healthcare characteristics	Yes	Yes	Yes
	Economic characteristics	Yes	Yes	Yes
	Regional characteristics	Yes	Yes	Yes
Constant		4.111^***^ (0.099)	0.173^***^ (0.062)	4.269^***^ (0.142)
*N*		5,287	5,287	5,287
*F*-statistic		26.99	88.64	—

Table 8 reports the results from alternative estimation methods. The coefficient for self-employment remains negative and statistically significant at the 10% level in both the OLS and IV-2SLS specifications, indicating that the negative association is robust to alternative model specifications.

#### Replacement of explained variable

Table 9 presents results using objective short-term morbidity indicators as the dependent variable (binary probit). Columns (1–5) correspond to fever/sore throat/cough, diarrhea, stomachache, headache/dizziness, and joint/muscle pain; Column (6) is a composite monthly morbidity indicator. Most coefficients are positive. The composite morbidity coefficient (Column 6) is positive and significant at the 5% level.

**Table 9:** Robustness test II: results of self-employment and short-term health outcomes.

Variables		(1)	(2)	(3)	(4)	(5)	(6)
Core explanatory variables	Self-employment (= 1)	−0.014 (0.063)	0.080 (0.110)	0.162^*^ (0.096)	0.017 (0.087)	0.136^*^ (0.079)	0.117^**^ (0.052)
Control variable	Individual characteristics	Yes	Yes	Yes	Yes	Yes	Yes
	Healthcare characteristics	Yes	Yes	Yes	Yes	Yes	Yes
	Economic characteristics	Yes	Yes	Yes	Yes	Yes	Yes
	Regional characteristics	Yes	Yes	Yes	Yes	Yes	Yes
Constant		−1.395^***^	−1.661^***^	−2.466^***^	−2.013^***^	−2.374^***^	−1.281^***^
		(0.221)	(0.376)	(0.372)	(0.359)	(0.380)	(0.200)
Observations		5,287	5,287	5,287	5,287	5,287	5,287
Pseudo R^2^		0.0312	0.0525	0.0427	0.0413	0.0574	0.0335

The symbols ^*^, ^**^, and ^***^ indicate statistical significance at the 10%, 5%, and 1% levels, respectively. Specifically, ^*^p < 0.10, ^**^p < 0.05, and ^***^p < 0.01.

Overall, the robustness checks using objective short-term morbidity indicators provide additional support for the adverse association between self-employment and health outcomes.

## Discussion and conclusion

### Principal findings

Overall, the empirical findings are consistent with the three proposed hypotheses. The results support the negative association between self-employment and health (Hypothesis 1), identify nutrition and lifestyle-related correlates as relevant correlates (Hypothesis 2), and reveal significant heterogeneity across different types of self-employment (Hypothesis 3). The robustness checks—including alternative estimators (OLS and IV-2SLS) and the replacement of the dependent variable with objective short-term morbidity indicators—further support the adverse association between self-employment and health. The composite morbidity indicator is positively and significantly associated with self-employment, corroborating the baseline finding based on self-rated health.

### Interpretation of the self-employment–health association

This study shows that self-employment is associated with poorer health outcomes among Thai adults. From a nutrition and lifestyle perspective, these findings suggest that employment arrangements are associated with health-related inequalities through differences in income stability, occupational status, dietary behaviors, daily routines, stress exposure, and access to preventive resources. Self-employed workers, especially solo self-employed individuals, often face unstable schedules and economic uncertainty, which may be associated with irregular eating patterns, reduced dietary diversity, lower physical activity, and delayed healthcare utilization.

A plausible interpretation of the negative association can be derived from Karasek's Job Demand-Control Model (51). Despite greater decision-making autonomy, the self-employed bear heavier workloads. The combination of high job demand and high job control can generate intense work pressure, associated with poorer physical and mental health. Two factors may help explain this association. First, self-employed individuals run businesses with their own capital and confront greater risks and challenges; following the rational economic man assumption and the pursuit of utility maximization, they tend to work intensively and maintain strong accountability for business operations. Second, self-employment is generally characterized by small operational scale and low entry barriers. Workers in this sector often act as price takers in competitive markets and must therefore continuously analyze market information, identify overlooked business opportunities, and explore development paths for their products and services.

The negative coefficient for healthcare utilization observed in the baseline model should not be interpreted as evidence that healthcare use worsens health. Because this variable captures whether respondents received healthcare services during the reference period, its negative sign more plausibly reflects health-need selection or reverse causality: individuals with poorer underlying health are more likely to seek care during the survey period.

### Heterogeneity: employer vs. solo self-employment

The subsample analyses reveal that employer self-employed individuals tend to experience better health outcomes, whereas solo self-employed workers suffer from deteriorated health, with both results statistically significant. Two main reasons may account for this discrepancy. Employer self-employed individuals may benefit from better resources and working conditions. Such entrepreneurial activities, characterized by modern entrepreneurship, bring favorable working conditions and higher income, associated with better health outcomes. In contrast, solo self-employed workers are often pushed into this occupation. Constrained by insufficient human capital, they face limited job opportunities and turn to self-employment merely as a means of making a living. Compared with voluntary entrepreneurship, subsistence self-employment entails lower earnings and poorer working environments, associated with worse health conditions. The marginal effects further corroborate this pattern: employer self-employment is associated with increased probabilities of good and very good health, whereas solo self-employment is associated with increased probabilities of poor and fair health. These findings highlight substantial within-group heterogeneity and underscore the importance of distinguishing between different forms of self-employment in health research and policy design.

### Nutrition pathway

Dietary diversity is a useful proxy for diet quality but is not equivalent to comprehensive nutritional status; the following interpretation should be read with this caveat in mind. Nutrition is a core determinant of health and a central component of lifestyle-related inequalities. A large body of nutritional epidemiology literature has demonstrated that dietary quality, including dietary diversity, is strongly associated with chronic disease risk, metabolic health, and overall mortality. Employment conditions may influence nutritional outcomes through income stability, time constraints, food environment accessibility, and work-related stress, all of which are key determinants in nutrition science. Dietary diversity has been widely validated in low- and middle-income country settings as a proxy indicator of micronutrient adequacy and overall diet quality (52, 53). Although it does not capture portion size, caloric intake, or detailed nutrient composition, it represents a practical and comparable measure for large-scale population surveys, consistent with FAO and WHO guidelines on dietary diversity measurement in resource-limited settings.

The finding that self-employment is negatively associated with dietary diversity suggests that self-employed individuals may have lower dietary diversity compared with paid employees. These findings contribute to the growing literature in nutritional epidemiology by highlighting the association between employment type and dietary behaviors and nutrition-related inequalities. From a food insecurity perspective, previous studies have shown that unstable income and labor market vulnerability are closely associated with reduced food security, lower dietary quality, and increased reliance on low-cost, energy-dense foods. Research on informal and non-standard workers further suggests that precarious employment conditions are associated with irregular eating patterns, reduced dietary diversity, and higher exposure to unhealthy food environments. These patterns are particularly relevant in low- and middle-income countries where informal employment is widespread. Furthermore, the food environment literature emphasizes that access to affordable and healthy food options is unevenly distributed, and individuals in economically vulnerable employment positions may face structural constraints that limit healthy dietary choices. In this context, self-employment may serve as an important socioeconomic correlate of nutrition inequality through both food insecurity and constrained food environments.

### Lifestyle pathway

Beyond nutrition, self-employment is also associated with other lifestyle behaviors, including lower physical activity and higher probabilities of smoking and alcohol consumption. Although not all coefficients reach conventional levels of statistical significance, the overall pattern is consistent with the theoretical expectation. These findings are consistent with Hypothesis 2 and suggest that lifestyle-related factors may be relevant correlates of both self-employment and health outcomes. However, given the cross-sectional nature of the data, these results should be interpreted as exploratory associations rather than evidence of mediation or causal pathways. This pattern is also consistent with the theoretical framework, which suggests that irregular working schedules, time constraints, and economic uncertainty may translate into unhealthy lifestyle behaviors among the self-employed. High job demands and long working hours are often linked to reduced physical activity due to limited leisure time and fatigue (38), while psychosocial stress associated with income instability may increase the likelihood of unhealthy coping behaviors such as smoking and alcohol consumption (39).

### Endogeneity and robustness

The consistency between OLS and IV estimates provides additional reassurance regarding the robustness of the baseline association. The larger IV coefficients may reflect the violation of the exclusion restriction rather than a true causal effect. The first-stage *F*-statistics (ranging from 72.89 to 88.64) well exceed the conventional threshold of 10, suggesting that the instrument is relevant and unlikely to suffer from weak-instrument problems. However, instrument relevance does not guarantee the validity of the exclusion restriction, which remains an identifying assumption. Because the exclusion restriction cannot be empirically verified, the IV estimates are interpreted as complementary sensitivity analyses rather than definitive causal estimates. The second-stage results remain significantly negative, broadly consistent with the baseline ordered probit association between self-employment and health.

### Policy implications

From a policy perspective, improving population health requires a stronger integration of labor market policies with nutrition- and lifestyle-oriented interventions. From a nutrition policy perspective, improving dietary quality requires addressing not only individual behaviors but also structural determinants such as income stability, food affordability, and accessibility within local food environments. Three policy directions are warranted. First, targeted nutrition support programs should be developed for vulnerable self-employed groups, particularly solo self-employed individuals, to improve dietary diversity and access to healthy foods. Second, public health strategies should promote flexible and accessible interventions, such as community-based nutrition education, healthy food environments, and workplace-independent health promotion programs that are suitable for individuals outside formal employment systems. Third, strengthening social protection systems, including health insurance coverage and preventive healthcare access, may indirectly improve nutrition outcomes by reducing economic insecurity and health-related constraints.

From a nutrition policy perspective, improving dietary quality requires addressing not only individual behaviors but also structural determinants such as income stability, food affordability, and accessibility within local food environments.

### Limitations

Several limitations of this study should be acknowledged when interpreting the results. First, the cross-sectional nature of the data limits our ability to establish causal relationships between self-employment and health outcomes. Reverse causality may exist, as health status can also influence individuals' employment choices. Second, although a rich set of control variables is included, residual confounding from unobserved factors such as personality traits, risk preferences, and early-life conditions cannot be ruled out. Third, the primary health outcome is based on self-rated health, which, while widely used in the literature, may be subject to subjective bias and reporting heterogeneity across individuals. Fourth, lifestyle-related variables such as dietary behaviors, physical activity, smoking, and alcohol consumption are self-reported and may be affected by measurement error or recall bias. Fifth, the nutritional analysis is limited to a single indicator—dietary diversity—derived from one 24-h recall using an adapted 10-group HDDS measure. This indicator does not assess energy intake, macronutrient or micronutrient adequacy, portion sizes, or overall dietary quality. It captures only the variety of food groups consumed and may not reflect usual dietary patterns. Future studies should incorporate repeated 24-h recalls, food frequency questionnaires, or biochemical markers to provide a more comprehensive nutritional assessment. Sixth, the instrumental-variable analysis is not sufficiently convincing for causal inference. The exclusion restriction requires that regional unemployment affects health only through self-employment, but this assumption is likely violated: regional unemployment may influence health through local economic conditions, food affordability, healthcare infrastructure, stress environments, and other channels. Therefore, the IV results should be interpreted strictly as sensitivity checks, not as causal estimates. Regional unemployment may affect health through multiple channels, including local economic conditions, healthcare accessibility, and food environments, rather than exclusively through self-employment status; therefore, the IV estimates should be interpreted as complementary evidence supporting the robustness of the baseline association rather than as definitive causal estimates. Seventh, while the Thailand Health and Welfare Survey employs a complex multi-stage sampling design, not all analyses fully incorporate survey weights and design features, which may affect the precision of standard errors. Finally, the cross-sectional framework limits our ability to formally identify mechanisms. Although lifestyle-related variables are explored as potential pathways, these analyses remain associative rather than causal or mediating in nature. Future research using longitudinal data would be better suited to establish causal pathways between employment type, lifestyle behaviors, and health outcomes.

## Conclusion

This study examines the association between self-employment and health outcomes among Thai adults, with a focus on nutrition and lifestyle behaviors. Using data from the 2024 Thailand Health and Welfare Survey and employing survey-adjusted ordered probit models with instrumental-variable sensitivity analysis, the results show that self-employment is associated with lower self-rated health, reduced dietary diversity, and less favorable lifestyle behaviors, including lower physical activity and higher probabilities of smoking and alcohol consumption. The associations are heterogeneous: employer self-employed individuals tend to maintain healthier behaviors and better health outcomes, whereas solo self-employed workers are more vulnerable to lower dietary diversity and adverse health conditions. These findings suggest that employment type is a structural factor associated with nutrition, lifestyle behaviors, and health inequalities. Policies aimed at improving population health should integrate labor market interventions with nutrition- and lifestyle-oriented strategies, particularly for vulnerable self-employed groups.

## Data Availability

The data analyzed in this study is subject to the following licenses/restrictions: contact the author to provide. Requests to access these datasets should be directed to Kai Yao, 506854753@qq.com.
