# Supplementary material for: Integrating nutrition and lifestyle: associations between employment type and health behaviors
Source: Front Nutr. 2026 Oct 16;13:1889959. doi: 10.3389/fnut.2026.1889959 (PMC13719413; doi:10.3389/fnut.2026.1889959)
Supplement: Supplementary file 1 [file Table_1.docx]

**Supplementary Material**

**Table S1. Definitions and coding of all variables used in the analyses**

| **Variable** | **Definition** |
| --- | --- |
| Dietary diversity | Number of food groups consumed |
| Physical activity | Regular activity=1 |
| Smoking | Current smoker=1 |
| Drinking | Current drinker=1 |
| Healthcare utilization | Received healthcare during reference period=1 |
